# Contraceptive knowledge and use among women living in the poorest areas of five Mesoamerican countries

**DOI:** 10.1016/j.contraception.2017.01.005

**Published:** 2017-06

**Authors:** Diego Rios-Zertuche, Laura C. Blanco, Paola Zúñiga-Brenes, Erin B. Palmisano, Danny V. Colombara, Ali H. Mokdad, Emma Iriarte

**Affiliations:** a Salud Mesoamérica Initiative/Inter-American Development Bank, Calle 50, Edificio Tower Financial Center (Towerbank), Piso 23, Panama, Panama; b Department of Economics, Universidad de Costa Rica; c Institute for Health Metrics and Evaluation, 2301 5th Ave, Suite 600, Seattle, WA, USA

**Keywords:** Health inequalities, Unmet need, Poverty, Contraceptive knowledge, Family planning, Salud Mesoamerica

## Abstract

**Objective:**

To identify factors associated with contraceptive use among women in need living in the poorest areas in five Mesoamerican countries: Guatemala, Honduras, Nicaragua, Panama and State of Chiapas (Mexico).

**Study design:**

We analyzed baseline data of 7049 women of childbearing age (15–49 years old) collected for the *Salud Mesoamérica Initiative*. Data collection took place in the 20% poorest municipalities of each country (July, 2012-August, 2013).

**Results:**

Women in the poorest areas were very poorly informed about family planning methods. Concern about side effects was the main reason for nonuse. Contraceptive use was lower among the extremely poor (<$1.25 USD PPP per day) [odds ratio (OR): 0.75; confidence interval (CI): 0.59–0.96], those living more than 30 min away from a health facility (OR 0.71, CI: 0.58–0.86), and those of indigenous ethnicity (OR 0.50, CI: 0.39–0.64). Women who were insured and visited a health facility also had higher odds of using contraceptives than insured women who did not visit a health facility (OR 1.64, CI: 1.13–2.36).

**Conclusions:**

Our study showed low use of contraceptives in poor areas in Mesoamerica. We found the urgent need to improve services for people of indigenous ethnicity, low education, extreme poverty, the uninsured, and adolescents. It is necessary to address missed opportunities and offer contraceptives to all women who visit health facilities. Governments should aim to increase the public's knowledge of long-acting reversible contraception and offer a wider range of methods to increase contraceptive use.

**Implications:**

We show that unmet need for contraception is higher among the poorest and describe factors associated with low use. Our results call for increased investments in programs and policies targeting the poor to decrease their unmet need.

## 1 Introduction

Despite generalized efforts to offer universal access, family planning is still among the most inequitable interventions for women in the poorest quintile of women [1]. Inequalities are particularly noticeable in Mesoamerica, an area encompassing the south of Mexico and Central America. Low contraceptive use and high fertility endure among indigenous, poor and rural populations [2], [3], [4], [5], [6], [7]. These countries are among the most inequitable in the world [8], [9] and have the highest levels of extreme poverty in Latin America [10]. Health systems in Mesoamerica are highly segmented, and while ministries of health serve rural areas, the poor, and the uninsured, funds are unequally distributed among institutions [2], [3], [4], [11], [12]. Not everyone has regular access to services. Ministries of health need to increase access to and use of effective contraception among the poor, which continues to be a central strategy to reduce maternal mortality [13].

We analyze factors associated with modern contraceptive use for women living in the poorest areas of Guatemala, Honduras, Nicaragua, Panama and the State of Chiapas in Mexico. We seek to provide information on contraceptive use to strengthen strategies and programs seeking to reduce the gap of unmet need.

## 2 Materials and methods

### 2.1 Data source

We analyze baseline data collected for *Salud Mesoamérica Initiative* (SMI), a results-based financing program supporting maternal and child health improvements [14]. Our household survey samples represent the poorest municipalities in five countries: Guatemala (April–August 2013), Honduras (January–June 2013), Chiapas (Mexico) (July–May 2013), Nicaragua (March–August 2013), and Panama (April–August 2013). Survey methodology has been explained in detail [15]. In summary, census segments were selected among poor municipalities, with probability proportional to size. Each segment had approximately 150 households, which were censused. A random sample was selected of 30 households with women 15–49 years old or children under five. Field staff conducted computer-assisted personal interviews in Spanish or in indigenous languages. Our analysis focused on married or partnered women of childbearing age in need of contraception. We obtained informed consent from all informants. Institutional review boards at the University of Washington, data collection agencies and Ministries of Health reviewed and approved the study.

### 2.2 Definition of women in need of contraception.

A woman has contraceptive needs if she is 15–49 years old and does not want to become pregnant or wants to postpone pregnancy for at least 2 years [16], [17]. Women who became pregnant in the previous 2 years, or were pregnant when surveyed but did not want to get pregnant, are counted as in need. Women who wanted a child within 2 years of the survey are classified as not in need.

### 2.3 Definition of women using contraception

Women are considered to be using contraception if they were using a modern contraceptive method at the time of the survey. Modern methods include permanent methods, short-acting hormonal methods (SAHM), long-acting reversible contraception (LARC), barrier methods (BARR), and emergency contraception. Knowledge of each method was asked individually, including a description of its use. Women who had not heard of any method or were using only traditional methods (such as postpartum amenorrhea, rhythm or withdrawal) are classified as nonusers.

We analyzed contraceptive knowledge, most frequently used methods and reasons for nonuse. Women reporting interruptions in the year preceding the survey or not using any method on the day of the interview were asked follow-up questions to identify reasons for interruption or nonuse.

### 2.4 Data analysis

We used SMI data to calculate contraceptive prevalence among those in need. We calculated population estimates for factors potentially associated with contraceptive use. Individual and household level characteristics included age, gravidity, fertility, abortions or still births, age at first pregnancy and delivery, and visit to a health facility in the previous 12 months. We considered indigenous ethnicity when the head of household reported speaking an indigenous language, or when the census or surveys were conducted in indigenous languages. We created a binary variable for knowledge of fertile period. We also included the woman's employment status (employed or paid for work the previous week), years of education, highest level of education, and health insurance. We used total household expenditures to calculate if women lived in a poor household, defined as poverty headcount ratio at $2 USD per day (in purchasing power parity terms, PPP), or in an extremely poor household, with poverty headcount ratio at $1.25 USD per day (PPP). Accessibility factors included living more than 30 min away from a health facility and receiving family planning advice at the health facility or from a community health worker. We report estimates for our pooled sample and for each country, and compared characteristics across countries using χ^2^ tests and Wald tests.

We used multivariate logistic regression to determine factors associated with contraceptive use among women in need. We selected covariates using backward elimination. Covariates were retained when the Wald test had a p-value<0.05. We performed the same analysis in our pooled sample and country by country. For the pooled sample, we included country as a fixed effect. We examined interactions to test if the effect of visiting a health facility and using contraception was modified by insurance, advice in the health facility, or being indigenous, and to test if being indigenous and using contraception was modified by receiving advice at a health facility or from the community health worker (CHW). To report interaction effects, we included the ratio of odd ratios and conditional odd ratios [18]. Only insurance modified the odds of visiting a health facility and contraceptive use. We compared our results using education completed and years of education, which did not affect our results. Finally, we excluded Chiapas from the analysis, where most respondents have health insurance, to test the association between contraceptive use and health insurance. The effect of the interaction did not remain after excluding Chiapas.

We checked for goodness-of-fit using the Hosmer & Lemeshow test [19] and performed link tests for model specification [20]. Our variable for living more than 30 min away from a health facility had 8% missing values; all others missed less than 5%. We analyzed observations with complete data. We used Stata SE 12.1 (StataCorp LP, College Station, TX) for the analyses and svy command to account for complex survey design. Only our final models are shown.

## 3 Results

### 3.1 Study population

Our pooled sample included 7049 observations (see Table 1). Modern contraceptive use varied widely across countries. Nicaragua had the highest coverage (82.2%–95%, CI: 78.3–85.5) and Panama the lowest (15.3%, CI: 10.3–22.1). The only variable that did not differ significantly between countries was abortions or still births. In all countries, most women had their first live birth between 15–19 years old. Less than 8% had health insurance, except for in Chiapas, where over 86% were insured. In Chiapas and Guatemala more than half of the population lived with less than $2 USD a day and more than 40% with less than $1.25 USD a day. Most women were not employed and did not complete their primary education. The majority of the population in Chiapas, Guatemala and Panama had indigenous ethnicity.

**Table 1:** Characteristics of women in the lowest income quintile in Mesoamerica (2012–2013).

	Pooled sample	Chiapas (Mexico)	Guatemala	Honduras	Nicaragua	Panama	p-Value
Observations	7049	2446	2061	903	937	702	
Modern contraceptive use	58.2	49.8	27.5	69.2	82.2	15.3	0.000⁎
	[55.4–60.9]	[45.4–54.2]	[23.8–31.5]	[62.6–75.0]	[78.3–85.5]	[10.3–22.1]	
Gravidity *(average)*	3.8	3.8	3.9	3.5	3.0	3.5	0.000⁎
	[3.6–4.1]	[3.6–4.1]	[3.7–4.1]	[3.2–3.8]	[2.8–3.3]	[3.3–3.8]	
Fertility *(average)*	3.5	3.8	3.8	3.3	2.9	3.5	0.000⁎
	[3.3–3.6]	[3.6–4]	[3.6–3.9]	[3.1–3.6]	[2.7–3.1]	[3.2–3.7]	
Had abortion or still birth	8.6	7.5	7.3	12.2	10.1	5.6	0.052
	[7.4–10.0]	[6.1–9.3]	[5.7–9.3]	[9.0–16.3]	[7.2–14.0]	[3.7–8.5]	
Age at first live birth							0.002⁎
No children	3.7	2.6	3.4	4.3	5.5	4.4	
	[2.8–4.8]	[1.8–3.9]	[2.4–4.9]	[2.2–8.3]	[3.2–9.1]	[2.5–7.6]	
15 years or less	3.7	3.4	2.0	1.9	4.9	9.4	
	[3.2–4.4]	[2.6–4.4]	[1.4–3.0]	[1.1–3.3]	[3.9–6.2]	[5.2–16.4]	
15–19 years	58.6	57.5	58.5	60.9	60.4	51.3	
	[56.1–61.0]	[54.1–60.8]	[54.8–62.1]	[55.0–66.5]	[54.5–66.1]	[45.3–57.3]	
20–24 years	26.0	27.0	28.3	27.8	23.0	24.1	
	[23.8–28.2]	[24.5–29.6]	[25.6–31.3]	[22.9–33.3]	[17.6–29.5]	[19.3–29.7]	
25 years or older	8.1	9.5	7.7	5.1	6.2	10.7	
	[6.9–9.3]	[7.7–11.8]	[6.1–9.7]	[3.4–7.4]	[4.6–8.4]	[7.2–15.8]	
Knows when one is more likely to get pregnant	9.0	9.1	3.5	3.7	13.0	0.0	0.000⁎
	[7.5–10.8]	[6.9–11.8]	[2.3–5.2]	[2.5–5.6]	[9.8–17.1]	0.0	
Poor household (less than $2 USD PPP per day)	62.2	71.3	66.6	59.6	46.9	45.5	0.000⁎
	[58.9–65.4]	[66.3–75.8]	[62.8–70.1]	[54.0–65.0]	[40.5–53.4]	[36.6–54.8]	
Extremely poor household (less than $1.25 USD PPP per day)	38.8	48.6	42.2	33.9	22.3	32.7	0.000⁎
	[35.6–42.1]	[43.3–54.0]	[38.5–45.9]	[28.9–39.2]	[17.6–27.7]	[23.7–43.1]	
Woman employed previous week	8.8	6.6	2.6	7.9	14.8	7.9	0.000⁎
	[7.1–10.9]	[4.6–9.5]	[1.6–4.3]	[4.5–13.4]	[10.8–19.9]	[5.2–11.9]	
Years of education *(average)*	5.0	5.0	2.9	5.0	5.6	5.6	
	[4.6–5.4]	[4.6–5.4]	[2.5–3.3]	[4.5–5.6]	[4.7–6.5]	[4.8–6.3]	
Highest education level attained:							0.000⁎
Did not complete primary education	51.3	48.5	76.2	48.9	50.7	41.0	
	[47.7–54.8]	[43.9–53.1]	[72.0–79.9]	[42.3–55.5]	[41.7–59.6]	[33.3–49.0]	
Completed primary education	37.3	42.3	18.1	41.7	31.5	47.7	0.000⁎
	[34.7–39.9]	[38.5–46.2]	[15.8–20.7]	[36.2–47.4]	[26.2–37.4]	[40.5–55.0]	
Completed secondary education or above	11.5	9.2	5.7	9.4	17.8	11.4	
	[9.1–14.3]	[6.9–12.3]	[3.9–8.1]	[6.0–14.5]	[11.7–26.2]	[8.0–15.8]	
Woman’s age							0.040⁎
15–19 years	8.7	6.8	9.6	9.1	11.8	6.4	
	[7.7–9.8]	[5.7–8.1]	[8.1–11.4]	[7.3–11.4]	[9.1–15.2]	[4.6–8.8]	
20–24 years	17.8	16.2	18.3	18.8	20.1	18.8	
	[16.4–19.3]	[14.5–18.0]	[16.5–20.4]	[15.9–22.1]	[16.7–24.1]	[13.5–25.5]	
25–29 years	17.9	17.9	17.3	18.3	18.2	16.5	
	[16.5–19.4]	[16.2–19.6]	[15.5–19.3]	[15.1–22.0]	[14.6–22.4]	[13.2–20.5]	
30–34 years	18.5	19.5	19.0	18.0	16.9	15.4	
	[16.9–20.2]	[17.1–22.2]	[16.6–21.7]	[14.7–21.9]	[14.0–20.3]	[11.7–20.1]	
35–39 years	16.3	17.6	13.4	14.9	15.2	17.4	
	[14.6–18.2]	[15.4–20.2]	[11.5–15.5]	[11.8–18.8]	[11.4–19.9]	[12.4–23.9]	
40–44 years	12.6	13.7	12.8	12.8	10.5	13.6	
	[11.1–14.3]	[11.8–15.9]	[10.6–15.3]	[9.5–16.9]	[7.1–15.1]	[10.2–18.0]	
45–49 years	8.2	8.3	9.5	8.1	7.3	11.9	
	[7.0–9.5]	[6.5–10.4]	[7.6–11.9]	[5.3–12.1]	[5.4–9.9]	[6.9–19.6]	
Indigenous ethnicity	54.8	75.8	83.3	0.0	23.0	95.4	0.000⁎
	[49.8–59.7]	[68.5–81.8]	[76.3–88.5]	0.0	[12.3–39.0]	[90.5–97.8]	
Received family planning advice at health facility	31.3	32.4	15.7	30.0	34.8	24.9	0.000⁎
	[29.0–33.7]	[28.9–36.1]	[12.8–19.0]	[26.1–34.3]	[30.1–39.9]	[19.0–31.9]	
Received family planning advice from community health worker	16.5	22.4	12.1	15.5	8.1	8.4	0.000⁎
	[14.7–18.5]	[19.1–26.1]	[9.4–15.6]	[12.2–19.4]	[6.2–10.4]	[5.9–12.0]	
Health facility more than 30 min away	25.0	21.1	25.3	31.2	30.2	24.0	0.046⁎
	[21.8–28.6]	[16.6–26.4]	[20.9–30.4]	[24.1–39.2]	[22.8–38.8]	[15.3–35.6]	
Visited health facility in past 12 months	51.6	48.6	30.9	53.3	62.9	44.7	0.000⁎
	[48.9–54.3]	[44.4–52.9]	[26.7–35.5]	[47.4–59.1]	[57.1–68.4]	[36.3–53.4]	
Has health insurance	46.3	86.2	7.8	0.6	3.7	7.7	0.000⁎
	[44.2–48.5]	[83.6–88.4]	[5.1–12.0]	[0.3–1.3]	[1.7–8.0]	[4.9–11.9]	

Values are survey-weighted percentages of variables associated with contraceptive use by women in need from the poorest areas; 95% confidence intervals in brackets.

⁎ p*<.05.*

### 3.2 Contraceptive knowledge

**Fig. 1:**
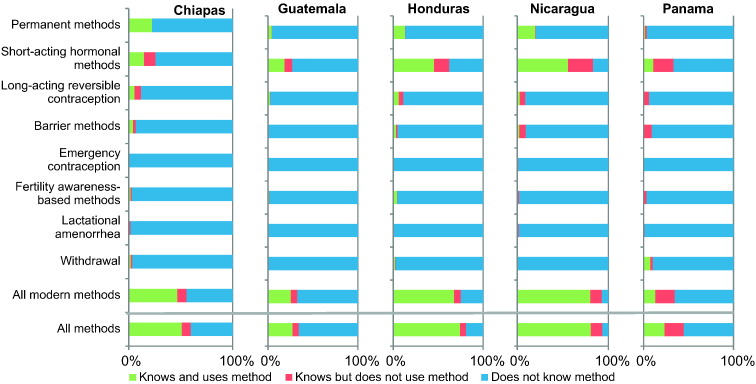
Knowledge about and use of contraceptive methods among women in the poorest areas in Mesoamerica (2012–2013). Survey-weighted knowledge and use of contraceptives among partnered women in need from the poorest areas. Modern methods include: permanent methods (male and female sterilization), short-acting hormonal methods (pill, injectables and vaginal ring), long-acting reversible contraception (implants and IUDs), barrier methods (male and female condoms, diaphragm, sponge), and emergency contraception. Traditional methods include: fertility awareness-based methods (rhythm) lactational amenorrhea, and withdrawal.

More than 30% of all women did not know any modern contraceptive (Fig. 1 and Table 2). On average, women knew less than two modern methods. SAHM were the most known, ranging from 83.3% (Nicaragua) to 25.7% (Chiapas). In all countries, traditional methods were reported by less than 6% of women. Emergency contraception was mostly unknown.

**Table 2:** Contraceptive knowledge and self-reported reasons for nonuse of modern contraceptives among poor women in Mesoamerica (2012–2013).

	Pooled sample	Chiapas (Mexico)	Guatemala	Honduras	Nicaragua	Panama	p-Value
Observations	7049	2446	2061	903	937	702	
Women who do not know any modern methods	32.3	41.4	65.4	23.8	6.1	67.4	0.000⁎
	[29.5–35.2]	[36.8–46.1]	[61.1–69.5]	[18.5–30]	[4–9.2]	[59.4–74.5]	
Number of contraceptives known *(average)* (all partnered women who know at least 1 method)	1.4	1.3	1.1	1.4	1.6	2.4	0.000⁎
	[1.4–1.5]	[1.2–1.3]	[1.1–1.2]	[1.3–1.5]	[1.5–1.7]	[2.1–2.6]	

Reasons for non-use among women in need of contraception (women could provide more than 1 option)							
Not married or having infrequent sex	8.2	6.4	6.7	16.3	14.8	7.7	0.338
	[6.3–10.6]	[4.5–9.0]	[4.9–9.1]	[10.4–24.6]	[6.4–30.6]	[4.8–12.2]	
Married	16.6	23.8	1.9	6.1	1.2	25.8	0.000⁎
	[14.0–19.7]	[19.7–28.4]	[1.1–3.3]	[3.2–11.2]	[0.2–8.2]	[16.8–37.5]	
Infertility issues	4.7	4.8	1.9	11.1	5.1	3.2	0.151
	[3.3–6.6]	[3.0–7.8]	[1.1–3.2]	[4.4–25.1]	[2.2–11.5]	[1.7–5.9]	
No period after last birth or breast feeding	7.8	9.1	3.9	5.0	7.4	6.8	0.000⁎
	[6.5–9.3]	[7.2–11.4]	[2.5–6.0]	[2.7–9.1]	[4.6–11.9]	[3.8–11.6]	
Opposed to birth control (religious–cultural–social reasons)	20.6	23.7	15.8	14.2	12.1	28.1	0.000⁎
	[18.0–23.6]	[20.0–27.8]	[12.8–19.4]	[7.9–24.1]	[5.5–24.4]	[20.1–37.8]	
Does not know where to get contraceptives	2.2	2.9	2.5	0.4	0	0.4	0.002⁎
	[1.4–3.4]	[1.6–5.0]	[1.7–3.7]	[0.1–3.2]		[0.1–1.2]	
Side-effects–uncomfortable–interferes with body–affects health–not like it	44.4	49.4	32.3	44.2	48.6	7.7	0.000⁎
	[41.0–47.9]	[44.6–54.2]	[28.4–36.4]	[34.0–54.8]	[35.7–61.7]	[4.8–12.0]	
Affordability (distance–cost–transportation)	1.5	1.5	1.0	0.7	2.1	2.1	0.197
	[1.0–2.2]	[0.9–2.5]	[0.6–1.7]	[0.2–3.2]	[0.6–7.1]	[0.9–4.8]	
Preferred method not available or no method available	1.2	1.7	0.2	0.5	0.6	0.8	0.005⁎
	[0.6–2.3]	[0.8–3.6]	[0.0–0.5]	[0.1–3.4]	[0.1–4.3]	[0.3–1.9]	
Hard to deal with or does not trust health facility staff	1.5	2.1	0.7	1.0	0.5	0.7	0.003⁎
	[0.8–2.8]	[1.0–4.3]	[0.4–1.2]	[0.3–2.8]	[0.1–3.5]	[0.2–2.1]	
Currently pregnant^1^	1.6	1.8	0.2	0.7	2.9	0.2	0.178
	[1.0–2.5]	[1.1–3.1]	[0.1–1.0]	[0.2–2.8]	[0.9–9.2]	[0.1–0.8]	
Other reason	6.4	4.6	7.1	10	13.5	3.2	0.057
	[4.9–8.2]	[3.0–7.1]	[5.3–9.5]	[5.3–18.0]	[7.6–22.8]	[1.5–6.5]	

Values are survey-weighted percentages or averages of contraceptive knowledge and reasons for not using modern contraception among women in need from the poorest areas.

1 Women who became pregnant in the previous two years or were pregnant when surveyed but did not want to get pregnant at the time of the survey were considered as being in need of contraception. This percentage shows cases of women who declared they were not using contraception due to their unintended pregnancy.

⁎ p*<.05.*

### 3.3 Types and source of contraceptives

**Fig. 2:**
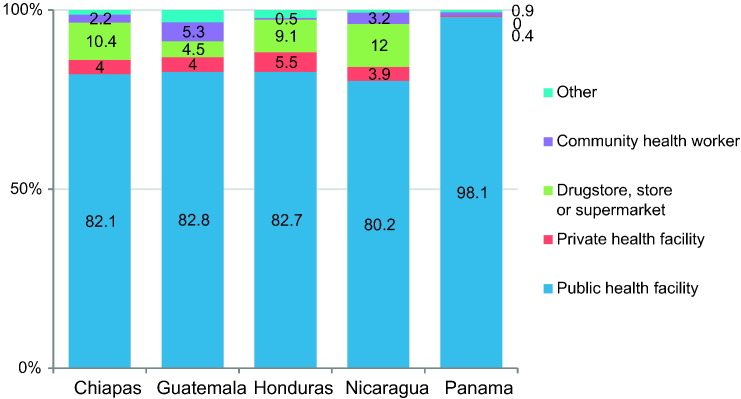
Source of family planning methods among women using contraception in Mesoamerica. Values are survey-weighted percentages for sources of family planning methods among women using contraception from the poorest areas.

In Guatemala, Nicaragua, Panama and Honduras, SAHM were the most commonly used (see Fig. 1). In Chiapas, permanent methods were the most frequently used, followed by SAHM. These two categories account for over 70% of all methods across countries, and LARC and BARR were used by 10% or less women. Over 80% of all women obtained their methods from public health facilities (Fig. 2).

### 3.4 Reasons for nonuse

The most commonly cited reason for contraceptive nonuse was health concerns (side effects, feeling uncomfortable), except for in Panama (see Table 2), where the main reason for nonuse was opposition to birth control (28.1% [20.1,37.8]), which was also important in all other countries.

### 3.5 Factors associated with contraceptive use

Women with more pregnancies (OR 0.92, CI: 0.87–0.97), those living far from a health facility (OR 0.75, CI: 0.59–0.96), and those from extremely poor households (OR 0.71, CI: 0.58–0.86) had lower odds of using contraceptives (see Table 3). In contrast, women who received advice at a health facility (OR 1.39, CI: 1.08–1.79), those who were employed (OR 1.48, CI: 1.03–2.13), and those with more years of education (OR 1.03, CI: 1.00–1.07) had higher odds. Indigenous ethnicity decreased the likelihood of contraceptive use by 50% (OR 0.50, CI: 0.39–0.64). Women 15–19 years old had the least odds for contraceptive use (OR 0.67, CI: 0.50–0.89) and women 30–34 years old had the highest (OR 1.56, CI: 1.16–2.10).

**Table 3:** Multivariate weighted odd ratios for using modern contraception for the poorest women in Mesoamerica.

Dependent variable: Modern contraceptive use (1 yes – 0 no)	Pooled sample	Chiapas (Mexico)	Guatemala	Honduras	Nicaragua	Panama
Observations	7049	2446	2061	903	937	702
Gravidity (continuous)	0.92⁎	0.89⁎	0.99	1.01	0.94	0.92
	[0.87–0.97]	[0.83–0.96]	[0.91–1.07]	[0.9–1.12]	[0.79–1.1]	[0.78–1.09]
Received advice on family planning at health facility	1.39⁎	1.42⁎	4.71⁎	0.90	1.08	5.65⁎
	[1.08–1.79]	[1.03–1.94]	[3.1–7.18]	[0.51–1.58]	[0.55–2.12]	[2.5–12.74]
Health facility more than 30 min away	0.75⁎	0.72⁎	0.55⁎	0.70	0.88	0.78
	[0.59–0.96]	[0.52–0.99]	[0.39–0.77]	[0.44–1.11]	[0.48–1.62]	[0.37–1.65]
Extremely poor household(less than $1.25 USD PPP per day)	0.71⁎	0.70⁎	0.60⁎	0.61⁎	0.81	0.59
	[0.58–0.86]	[0.54–0.92]	[0.42–0.85]	[0.38–0.99]	[0.49–1.35]	[0.28–1.24]
Woman employed previous week	1.48⁎	1.04	0.36⁎	1.76	3.45⁎	1.14
	[1.03–2.13]	[0.62–1.74]	[0.14–0.97]	[0.61–5.07]	[1.61–7.4]	[0.39–3.28]
Years of education (continuous)	1.03⁎	1.05⁎	1.09⁎	1.01	1.01	1.14⁎
	[1–1.07]	[1.01–1.09]	[1.05–1.13]	[0.94–1.08]	[0.92–1.11]	[1.06–1.23]
Woman’s age						
15–19 years	0.67⁎	0.41⁎	0.43⁎	1.44	0.97	0.45
	[0.5–0.89]	[0.27–0.65]	[0.25–0.74]	[0.66–3.14]	[0.41–2.32]	[0.14–1.47]
20–24 years	0.91	0.79	0.73	1.11	1.43	0.54
	[0.72–1.15]	[0.59–1.06]	[0.47–1.14]	[0.6–2.05]	[0.58–3.49]	[0.19–1.56]
Reference: 25–29 years	1.00	1.00	1.00	1.00	1.00	1.00
30–34 years	1.56⁎	2.38⁎	1.22	0.89	0.67	0.60
	[1.16–2.1]	[1.67–3.39]	[0.78–1.92]	[0.46–1.74]	[0.3–1.51]	[0.23–1.58]
35–39 years	1.33	1.67⁎	1.26	0.78	0.86	2.50
	[0.96–1.85]	[1.07–2.63]	[0.79–2.01]	[0.36–1.7]	[0.38–1.96]	[0.76–8.24]
40–44 years	0.98	1.63⁎	0.79	0.64	0.30⁎	2.24
	[0.67–1.45]	[1.04–2.56]	[0.44–1.43]	[0.24–1.7]	[0.1–0.85]	[0.74–6.79]
45–49 years	0.81	1.28	0.25⁎	0.45	0.37	0.80
	[0.5–1.32]	[0.69–2.38]	[0.1–0.59]	[0.12–1.71]	[0.1–1.42]	[0.19–3.31]
Indigenous ethnicity	0.50⁎	0.57⁎	0.91	(omitted)	0.34⁎	0.11⁎
	[0.39–0.64]	[0.42–0.77]	[0.6–1.37]		[0.21–0.57]	[0.03–0.4]
Visited health facility in the past 12 months	(interaction)	0.69⁎	0.83	1.47	1.09	2.30⁎
		[0.5–0.95]	[0.56–1.21]	[0.78–2.8]	[0.57–2.07]	[1.18–4.51]
Has health insurance	(interaction)	1.72⁎	1.70	(omitted)	7.64	1.48
		[1.18–2.49]	[0.99–2.9]		[0.54–107.35]	[0.55–3.98]
Visited health facility x Has health insurance	0.50⁎	(omitted)	(omitted)	(omitted)	(omitted)	(omitted)
	[0.34–0.73]					
Not insured, visited a health facility vs. did not	1.29					
	[0.96–1.74]					
Insured– visited a health facility vs. did not	0.65⁎					
	[0.46–0.89]					
Did not visit a health facility– insured vs. not insured	2.55⁎					
	[1.76–3.69]					
Visited a health facility– insured vs. not insured	1.28					
	[0.89–1.79]					
Insured and visited vs. not insured and did not visit	1.64⁎					
	[1.13–2.36]					
Reference: Mexico	1.00	(omitted)	(omitted)	(omitted)	(omitted)	(omitted)
Guatemala	0.72	(omitted)	(omitted)	(omitted)	(omitted)	(omitted)
	[0.51–1.02]					
Honduras	2.24⁎	(omitted)	(omitted)	(omitted)	(omitted)	(omitted)
	[1.43–3.51]					
Nicaragua	4.82⁎	(omitted)	(omitted)	(omitted)	(omitted)	(omitted)
	[3.19–7.27]					
Panama	0.28⁎	(omitted)	(omitted)	(omitted)	(omitted)	(omitted)
	[0.17–0.46]					
F statistic	25.97	7.95	8.62	1.70	2.55	6.83
Probability > F	0.00	0.00	0.00	0.10	0.02	0.00
H&L gof: prob. > F	0.18	0.44	0.19	0.84	0.34	0.07
Link test: ŷ^2^ p = value	0.08	0.12	0.82	0.42	0.72	0.85

Survey-weighted odd ratios with 95% confidence intervals in brackets. Hosmer and Lemeshow goodness-of-fit (H&L gof).

⁎ p*<.05.*

There is evidence that visiting a health facility and contraceptive use are modified by insurance. For women who visited a health facility, being insured did not affect contraceptive use. However, women who did not visit a health facility and were insured had higher odds of using contraceptives than those who were not (OR 2.55, CI: 1.76–3.69). Women who were insured and visited a health facility had also higher odds of using contraceptives than those not insured who did not visit a health facility (OR 1.64, CI: 1.13–2.36]). Nevertheless, among the insured, visiting a health facility had lower odds of contraceptive use (OR 0.65, CI: 0.46–0.89). When looking at country-specific samples, health insurance increases odds of contraceptive use only in Chiapas (OR 1.76, CI: 1.18–2.49), while visiting a health facility reduces its likelihood (OR 0.69, CI: 0.50–0.95). Indigenous ethnicity was the only significant covariate across countries (except Honduras) decreasing the odds of contraceptive use.

### 3.6 Comparison with national surveys

Contraceptive coverage in the poorest areas was substantially lower than national averages, except for in Nicaragua (see Table 4). In most countries, estimates were also lower when compared to classification by rural residency, indigenous ethnicity or income quintile.

**Table 4:** Comparison of contraceptive prevalence rate between women in the poorest quintile and national survey estimates.

Country	SMI survey^1^	National surveys^2^			
	in poorest areas	National average	Poorest quintile	Rural residents	Indigenous ethnicity
Chiapas, Mexico	49.8	58.6			
Guatemala	27.5	65.4	48.1		52.2
Honduras	69.2	76.1	68.0	73.6	
Nicaragua	82.2	77.3		75.3	
Panama	15.3	71.5		74.6	26.1

1 Salud Mesoamerica Initiative (SMI) baseline surveys 2012–2013.

2 Chiapas, Mexico: Estimaciones de CONAPO para Chiapas de la Encuesta Nacional de la Dinámica Demográfica 2014 (ENADID). Guatemala: VI Encuesta Nacional de Salud Materno Infantil 2014–2015 (ENSMI). Honduras: Encuesta Nacional de Salud y Demografía 2011–2012 (DHS/ENDESA). Nicaragua: Encuesta Nicaragüense de Demografía y Salud 2011–2012 (DHS/ENDESA). Panama: Encuesta Nacional de Salud Sexual y Reproductiva Panama 2009 (ENASSER).

## 4 Discussion

Our study revealed that contraceptive use was substantially lower in the poorest areas compared to national averages. We found that indigenous ethnicity, extreme poverty, low education and living far from health facilities were associated with decreased contraceptive use. In contrast, health insurance and counseling at a health facility were associated with increased use. Our findings call for increased efforts to reach women in poor areas and provide the needed support to increase contraceptive use.

We found important differences in contraceptive use between countries. In the past two decades, the ministries of health of Honduras and Nicaragua implemented strong family planning programs supported by international funds [7]. In both countries, important efforts were undertaken to improve contraceptive supply chains and information flows to increase contraceptive use in rural areas [3], [21], [22]. Additionally, in Nicaragua, the healthcare delivery model stresses community participation and reinforces the community distribution of contraceptives in hard-to-reach areas [4], [23]. Although development aid for family planning programs has fallen significantly in the region [7], [13], strategic international investments could incentivize governments to address unmet needs in the poorest areas.

Our findings underscore that counseling at health facilities is a key intervention to satisfy unmet need, especially in countries with low prevalence rates (Chiapas, Guatemala and Panama). These findings are consistent with literature highlighting the critical role of information in the promotion of contraceptive use [24], [25]. Nevertheless, visiting a health facility per se does not imply that a woman in need would receive counseling. It is important to address missed opportunities to ensure all women with unmet needs are offered contraceptives.

Furthermore, contraceptives were highly unknown, and women have few methods to choose from. There is an opportunity to increase the use of LARC, which have been found to be highly cost-effective [26] and safe across all age-groups [27]. Although intrauterine devices (IUD) are freely and generally available in these countries, health providers do not always offer them [28] and rumors discourage use [28], [29]. Unfortunately, supply-targeted interventions have not effectively improved uptake [29]. The recent introduction of contraceptive implants opens a new possibility for poor women. The simpler insertion procedure and long-acting effects may encourage increased promotion and use. More needs to be known about the implant's acceptability and continued use among poor populations.

In Chiapas, *Seguro Popular* (SP) may be increasing access to contraception via immediate postpartum provision after in-facility births [30]. SP has been shown to be associated with in-facility birth [31] and antenatal care [33]. In the other three countries, where only employment-based or private insurance are available, we found no effect. In these countries, the growing focus of public health insurance on health promotion, rather than curative care, may increase contraceptive use.

Moreover, despite higher levels of insurance and the positive relationship of insurance and contraceptive use in Chiapas, visiting a health facility was negatively associated with contraceptive use. This finding may be another indicator of the practice of in-hospital postpartum contraception, or highlight the low emphasis on contraception by the *Oportunidades/Prospera* conditional cash transfer program, which targets poor women in Mexico. Previous work indicates that the program had no effect on adolescent women's use of modern contraceptive methods [32]. Nevertheless, more information is needed to explain the negative association between contraceptive use and health facility visits in Chiapas.

Consistent throughout the study, indigenous ethnicity was negatively associated with contraceptive use. People with indigenous ethnicity may face additional barriers to access, such as communication problems and discrimination. Doctors serving the poor often come from different cultural backgrounds and speak different languages from those they serve [33]. Improving cultural-awareness training, addressing stereotypes and discrimination, adapting counseling strategies, and supporting the development of a local health workforce could promote increased use. Addressing unmet needs of women who do not oppose contraception first may help lift cultural barriers and encourage others.

Our study showed that adolescents were less likely to use contraception. Young women have limited access to quality sexual and reproductive health services [34] and their contraceptive needs are not always effectively addressed [27]. In these areas, it is particularly important to encourage school attendance beyond primary school and integrate a curriculum of sexual and reproductive health [35].

Unfortunately, we were unable to collect data from women younger than 15, who are also at risk of pregnancy. Moreover, we had to limit our questions on contraceptive use to partnered or married women. Contraception is still a controversial topic, and obtaining responses was sometimes challenging [15].

Our study had other limitations. First, our data was self-reported and subject to social desirability bias. Second, we did not include objective measures for availability of methods, which could explain interruptions and nonuse. Third, we did not assess counseling quality and provider characteristics (such as gender, ethnicity and education level), which could further affect counseling. Finally, our study was cross-sectional, so we could not determine causality. However, our study is based on a large sample size and used a standard methodology, allowing comparisons across countries.

Our study showed wide disparities in contraceptive use within and between countries in Mesoamerica. We found the urgent need to improve services for people with indigenous ethnicity, low education, living in extreme poverty, the uninsured, and adolescents. It is also necessary to address missed opportunities and offer contraceptives to women in every visit. We found that *Seguro Popular* was indirectly associated with higher contraceptive use. Governments should aim to increase knowledge of LARC and offer a wider range of methods. Our results call for increased investments in programs and policies to decrease unmet need in these populations.
